# Fluorescence-based tools to improve biopharmaceutical process development

**DOI:** 10.1186/1753-6561-5-S8-O5

**Published:** 2011-11-22

**Authors:** Tiago M Duarte, Manuel JT Carrondo, Paula M Alves, Ana P Teixeira

**Affiliations:** 1 Instituto de Biologia Experimental e Tecnológica (IBET), Apartado 12, 2781-901 Oeiras, Portugal; 2 Instituto de Tecnologia Química e Biológica – Universidade Nova de Lisboa (ITQB-UNL), Apartado 127, 2781-901 Oeiras, Portugal

## Background

Process optimization and control are essential to match biopharmaceutical market demands. Early-steps in the development of new processes require screening hundreds of transfected clones in order to select those with the best expression characteristics, and identifying optimal environmental conditions for these clones to grow and express the protein of interest. While some culture systems include *in situ* analysis of cell density, most often by optical density or capacitance measurements, the majority of them still rely on off-line, laborious and time-consuming protocols for recombinant protein analysis. Spectroscopic methods have been proposed in literature to support bioprocesses development, because they are non-invasive and provide data on multiple components present in the culture bulks, thereby increasing information on process performance [1]. Here, we developed a fluorescence-based method for real-time monitoring of viable cells and antibody titers in bioreactor cultures, and extended this strategy for high throughput analysis of cellular productivity in 96-well plates.

## Materials and methods

For high-throughput cellular productivity analysis, three CHO-K1 cell clones with different IgG_4_ monoclonal antibody specific productivities were grown in 125mL shake flasks. Cultures were sampled twice a day to assess cellular growth; cell supernatants were stored at -20°C for further antibody quantification and fluorescence analysis. The supernatant samples were distributed into black 96-well plates and the fluorescence maps were collected in a spectrofluorometer (Horiba Jobin Yvon) connected to a microwell plate reader via optical fibers, placed above the microtiter plate [2]. For real-time monitoring of bioreactor cultures, the same three clones were grown in 650mL working volume bioreactors. Fluorescence analysis was performed *in situ*, with the optical fibres placed inside a submerged stainless steel probe, incorporating a quartz lens at the bottom.

Both excitation and emission slit widths were set equal to 5 nm and integration time was set at 0.5 sec. Fluorescence excitation-emission matrix, also called fluorescence map, was recorded in the excitation range of 285 - 485 nm, with a step of 20 nm, and in the emission range of 305 - 565 nm, with steps of 10 nm, giving a total of 176 excitation/emission wavelength pairs (λ_ex_/λ_em_). The acquisition time of each map is approximately 4.5 min. Further details are described in the literature [2,3].

## Results

**Figure 1:**
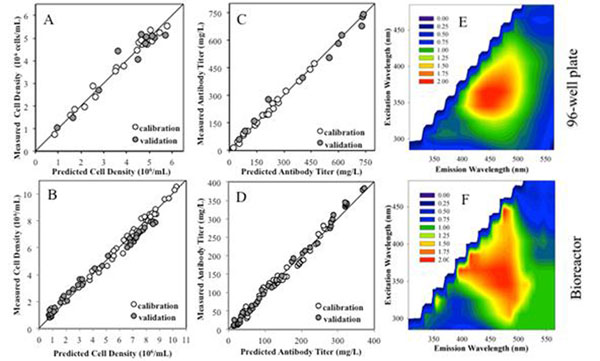
Correlations between predicted and measured cell density (A, B) or antibody titer (C, D), in 96-well plates or bioreactor cultures, as indicated. Open circles correspond to calibration data points and full circles correspond to validation data points. The optimum number of latent variables was selected such that the root mean squared error observed in the validation data set was minimum. For cell density, the best model structures are composed by 6 or 7 latent variables (LVs), corresponding to average errors of 10% or 9%, in the microtiter plates or bioreactor cultures, respectively. Larger models of 9 LVs allowed the best description for antibody titer, with average errors below 7%. Contour plots show the relative fluorescence changes in the map during a culture, analyzed in (E) 96-well plates and (F) in bioreactors. Colors reflect the magnitude of the ratio between maps from the end of a CHO cell culture and the beginning of the same culture.

The applicability of two dimensional (2D) fluorometry was studied using three CHO cell clones expressing an IgG_4_ to monitor viable cell density and antibody titer in bioreactor cultures and also in 96-well plates, targeting high throughput screening of cellular productivity. For the bioreactor application, the fluorescence analysis of the culture bulk was performed in situ, whereas for the 96-well plate application the cultures were performed in shake flasks and only the cell supernatant (without cells) was analyzed in terms of fluorescence. The three cell clones grow at similar rates and reach similar maximum cell densities but produce significantly different antibody quantities. Comparing bioreactor to shake flask cultures, all cell clones reached higher cell densities and lower antibody titers in the former system (Figure 1A-D).

As a result of cell growth and metabolism, significant changes were observed in all fluorescent regions. The global relative changes can be seen in Figure 1E-F, where it is plotted the ratios between fluorescence maps collected at late culture stages and maps collected after inoculation, in both culture systems under study. The larger changes occurred in the region of the cellular cofactors NAD(H)P (maximum at λ_ex_/λ_em_: 365/455nm), which increased significantly over culture time. In contrast, the fluorescence in the regions of tryptophan (maximum at λ_ex_/λ_em_: 285/365nm) and flavins (maximum at λ_ex_/λ_em_: 465/520) decreased along culture.

As the excitation light or the light emitted by fluorophors can be absorbed or dispersed by cells or other medium components, the signal that reaches the detector does not correlate linearly with the fluorophor concentration. Related to this, no linear correlations could be obtained between individual λ_ex_/λ_em_ pairs and our target bioprocess variables. Therefore, we adopted a multivariate statistical method, partial least squares, to construct the regression models linking the variation found in the spectra with the off-line measurements of the target variables. The dataset from both culture systems was split in two parts: one for calibration and the other for model validation. Both variables were predicted with good accuracies in the two configurations under study (Figure 1A-D). Importantly, the antibody profiles in the validation data sets could be described with an average error below 7%. This is a very good result, particularly because the validation data sets include concentrations above the ranges used to calibrate the models, revealing the extrapolation potential of the developed models.

## Conclusions

Both cell density and secreted antibody could be predicted with good accuracies in culture supernatant samples, demonstrating the potential of the method to effectively analyze cellular productivity in 96-well plate format. This methodology allows to by-pass the time-consuming ELISA assays and thus contributing to shorten early-phases of process development. The same approach was successfully implemented for *in-situ*, real-time monitoring of viable cells and antibody titer in bioreactor cultures, allowing continuous evaluation of bioprocess performance.
